# Photoinduced Topological Phase Transitions in Topological Magnon Insulators

**DOI:** 10.1038/s41598-018-22779-8

**Published:** 2018-03-13

**Authors:** S. A. Owerre

**Affiliations:** Perimeter Institute for Theoretical Physics, 31 Caroline St. N., Waterloo, Ontario, N2L 2Y5 Canada

## Abstract

Topological magnon insulators are the bosonic analogs of electronic topological insulators. They are manifested in magnetic materials with topologically nontrivial magnon bands as realized experimentally in a quasi-two-dimensional (quasi-2D) kagomé ferromagnet Cu(1–3, bdc), and they also possess protected magnon edge modes. These topological magnetic materials can transport heat as well as spin currents, hence they can be useful for spintronic applications. Moreover, as magnons are charge-neutral spin-1 bosonic quasiparticles with a magnetic dipole moment, topological magnon materials can also interact with electromagnetic fields through the Aharonov-Casher effect. In this report, we study photoinduced topological phase transitions in intrinsic topological magnon insulators in the kagomé ferromagnets. Using magnonic Floquet-Bloch theory, we show that by varying the light intensity, periodically driven intrinsic topological magnetic materials can be manipulated into different topological phases with different sign of the Berry curvatures and the thermal Hall conductivity. We further show that, under certain conditions, periodically driven gapped topological magnon insulators can also be tuned to synthetic gapless topological magnon semimetals with Dirac-Weyl magnon cones. We envision that this work will pave the way for interesting new potential practical applications in topological magnetic materials.

## Introduction

Topological insulators have captivated the attention of researchers in recent years and they currently represent one of the active research areas in condensed matter physics^1–5^. These nontrivial insulators can be realized in electronic systems with strong spin-orbit coupling and a nontrivial gap in the energy band structures. They also possess Chern number or ${{\mathbb{Z}}}_{2}$ protected metallic edge or surface modes that can transport information without backscattering^5^. In principle, however, the ubiquitous notion of topological band theory is independent of the statistical nature of the quasiparticle excitations. In other words, the concept of Berry curvature and Chern number can be defined for any topological band structure irrespective of the quasiparticle excitations. Consequently, these concepts have been extended to bosonic systems with charge-neutral quasiparticle excitations such as magnons^6–22^, triplons^23,24^, phonons^25,26^, and photons^27^.

Topological magnon insulators^6–17^ are the bosonic analogs of electronic topological insulators. They result from the nontrivial low-energy excitations of insulating quantum magnets with spin-orbit coupling or Dzyaloshinskii-Moriya (DM) interaction^28,29^, and exhibit topologically nontrivial magnon bands and Chern number protected magnon edge modes, with similar properties to those of electrons in topological insulators^1–5^. Theoretically, topological magnetic excitations can arise in different lattice geometries with DM interaction, however their experimental observation is elusive in real magnetic materials. Recently, intrinsic topological magnon insulator has been observed experimentally in a quasi-2D kagomé ferromagnet Cu(1–3, bdc)^16^. Moreover, recent evidence of topological triplon bands have also been reported in a dimerized quantum magnet SrCu_2_(BO_3_)_2_^23,24^. These magnetic materials have provided an interesting transition from electronic to bosonic topological insulators.

Essentially, the intrinsic properties of a specific topological magnon insulator are material constants that cannot be tuned, thereby hindering a topological phase transition in the material. In many cases of physical interest, however, manipulating the intrinsic properties of topological magnetic materials could be a stepping stone to promising practical applications, and could also provide a platform for studying new interesting features such as photo-magnonics^30^, magnon spintronics^31,32^, and ultrafast optical control of magnetic spin currents^33–36^. One way to achieve these scientific goals is definitely through light-matter interaction induced by photo-irradiation. In recent years, the formalism of photo-irradiation has been a subject of intensive investigation in electronic systems such as graphene and others^37–66^. Basically, photo-irradiation allows both theorists and experimentalists to engineer topological phases from trivial systems and also induce photocurrents and phase transitions in topologically nontrivial systems. In a similar manner to the notion of bosonic topological band theory, one can also extend the mechanism of photo-irradiation to bosonic systems.

In this report, we theoretically investigate photo-irradiated intrinsic topological magnon insulators in the kagomé ferromagnets and their associated topological phase transitions. One of the main objectives of this report is to induce tunable parameters in intrinsic topological magnon insulators, which subsequently drive the system into a topological phase transition. We achieve this objective by utilizing the quantum theory of magnons, which are charge-neutral spin-1 bosonic quasiparticles and carry a magnetic dipole moment. Therefore, magnons can couple to both time-independent^67–70^ and periodic time-dependent (see Methods)^71^ electric fields through the Aharonov-Casher (AC) effect^72^, in the same manner that electronic charged particles couple through the Aharonov-Bohm (AB) effect^73^. Quite distinctively, for the periodic time-dependent electric fields (see Methods)^71^, this results in a periodically driven magnon system, and thus can be studied by the Floquet-Bloch theory. Using this formalism, we show that intrinsic topological magnon insulators can be tuned from one topological magnon insulator to another with different Berry curvatures, Chern numbers, and thermal Hall conductivity. Moreover, we show that, by manipulating the light intensity, periodically driven intrinsic topological magnon insulators can also transit to synthetic gapless topological magnon semimetals. Therefore, the magnon spin current in topological magnetic materials can be manipulated by photo-irradiation, which could be a crucial step towards potential practical applications.

## Results

### Topological magnon insulators

We consider the simple microscopic spin Hamiltonian for intrinsic topological magnon insulators in the kagomé ferromagnets^16^

1 $$ {\mathcal H} =\sum _{\langle \ell \ell ^{\prime} \rangle }[-J{\overrightarrow{S}}_{\ell }\cdot {\overrightarrow{S}}_{\ell ^{\prime} }+{\overrightarrow{D}}_{\ell \ell ^{\prime} }\cdot ({\overrightarrow{S}}_{\ell }\times {\overrightarrow{S}}_{\ell ^{\prime} })]-\overrightarrow{B}\cdot \sum _{\ell }{\overrightarrow{S}}_{\ell }.$$

**Figure 1:**
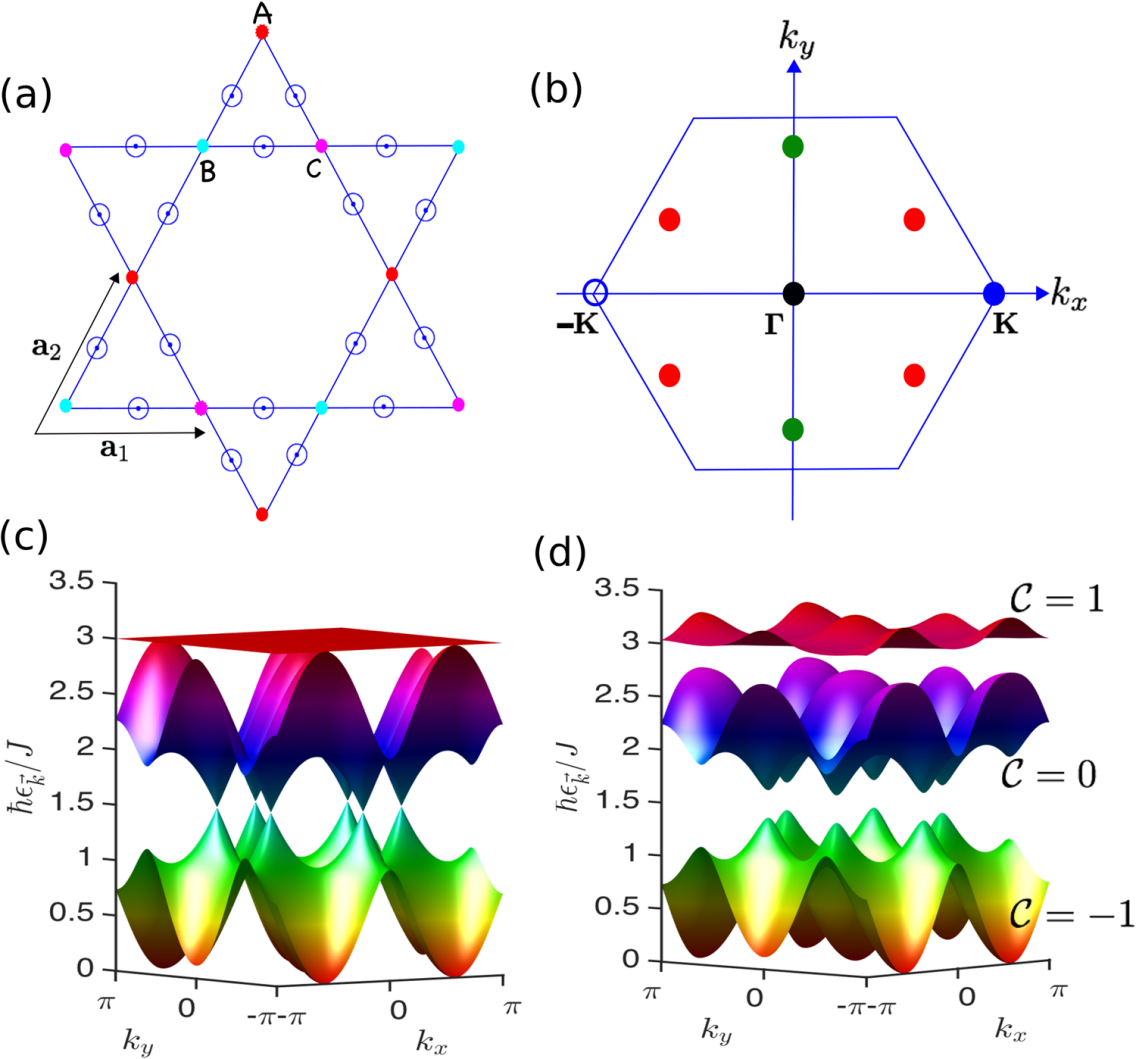
(**a**) Schematic of the kagomé lattice with three sublattices A, B, C as indicated by coloured dots, and the out-of-plane DM interaction is indicated by open circles. (**b**) The first Brillouin zone of the kagomé lattice with two inequvilaent high symmetry points at ±**K**. The red and green dots denote the photoinduced Dirac-Weyl magnon nodes as will be discussed later. (**c**) Magnon bands of undriven insulating kagomé ferromagnets with *D*/*J* = 0, showing Dirac magnon nodes at ±**K**, formed by the two lower dispersive bands. (**d**) Topological magnon bands of undriven insulating kagomé ferromagnets with *D*/*J* = 0.15.

The first summation is taken over nearest-neighbour (NN) sites $\ell$ and $\ell ^{\prime}$ on the 2D kagomé lattice, and ${\overrightarrow{D}}_{\ell \ell ^{\prime} }$ is the DM vector between the NN sites due to lack of an inversion center as depicted in Fig. (1)a. The last term is the Zeeman coupling to an external magnetic field $\overrightarrow{B}=g{\mu }_{B}\overrightarrow{H}$, where *μ*_*B*_ is the Bohr magneton and *g* the spin *g*-factor. Topological magnon insulators^6–8,10–17^ can be captured by transforming the spin Hamiltonian to a bosonic hopping model using the Holstein-Primakoff (HP) spin-boson transformation. In this formalism, only the DM vector parallel to the magnetic field contributes to the noninteracting bosonic Hamiltonian^6,16^, but other components of the DM vector can be crucial when considering magnon-magnon interactions^19^. Here, we limit our study to noninteracting magnon system as it captures all the topological aspects of the system^6,16^. We consider then an external magnetic field along the *z* (out-of-plane) direction, $\overrightarrow{B}=B\hat{z}$, and take the DM vector as ${\overrightarrow{D}}_{\ell \ell ^{\prime} }=D\hat{z}$. The Holstein-Primakoff (HP) spin-boson transformation is given by ${S}_{\ell }^{z}=S-{a}_{\ell }^{\dagger }{a}_{\ell },\,{S}_{\ell }^{+}\approx \sqrt{2S}{a}_{\ell }={({S}_{\ell }^{-})}^{\dagger }$, where ${a}_{\ell }^{\dagger }({a}_{\ell })$ are the bosonic creation (annihilation) operators, and ${S}_{\ell }^{\pm }={S}_{\ell }^{x}\pm i{S}_{\ell }^{y}$ denote the spin raising and lowering operators. Applying the transformation to Eq. (1) yields the bosonic (magnon) hopping Hamiltonian

2 $$ {\mathcal H} =-{t}_{0}\sum _{\langle \ell \ell ^{\prime} \rangle }({a}_{\ell }^{\dagger }{a}_{\ell ^{\prime} }{e}^{i{\phi }_{\ell \ell ^{\prime} }}+{\rm{H}}{\rm{.c}})+{t}_{z}\sum _{\ell }{n}_{\ell },$$

where ${n}_{\ell }={a}_{\ell }^{\dagger }{a}_{\ell }$ is the number operator; ${t}_{0}=JS\sqrt{1+{(D/J)}^{2}}$ and ${t}_{z}=4JS+B$ with ${t}_{z} > {t}_{0}$. The phase ${\phi }_{\ell \ell ^{\prime} }=\pm \phi =\pm {\tan }^{-1}(D/J)$ is the fictitious magnetic flux in each unit triangular plaquette of the kagomé lattice^6^, in analogy to the Haldane model^1^. The Fourier transform of the magnon Hamiltonian is given by ${\mathcal H} ={\sum }_{\overrightarrow{k}}{\psi }_{\overrightarrow{k}}^{\dagger } {\mathcal H} (\overrightarrow{k}){\psi }_{\overrightarrow{k}}$, with ${\psi }_{\overrightarrow{k}}={({a}_{\overrightarrow{k}\mathrm{,1}},{a}_{\overrightarrow{k}\mathrm{,2}},{a}_{\overrightarrow{k}\mathrm{,3}})}^{{\rm{T}}}$, where ${\mathcal H} (\overrightarrow{k})={t}_{z}{{\rm{I}}}_{3\times 3}-{\rm{\Lambda }}(\overrightarrow{k})$,

3 $${\rm{\Lambda }}(\overrightarrow{k})=2{t}_{0}(\begin{array}{ccc}0 & \cos \,{k}_{2}{e}^{-i\phi } & \cos \,{k}_{3}{e}^{i\phi }\\ \cos \,{k}_{2}{e}^{i\phi } & 0 & \cos \,{k}_{1}{e}^{-i\phi }\\ \cos \,{k}_{3}{e}^{-i\phi } & \cos \,{k}_{1}{e}^{i\phi } & 0\end{array}),$$

with ${k}_{i}=\overrightarrow{k}\cdot {\overrightarrow{a}}_{i}$, and ${\overrightarrow{a}}_{1}=\mathrm{(1},\mathrm{0)}$, ${\overrightarrow{a}}_{2}=(\mathrm{1/2},\sqrt{3}\mathrm{/2})$, ${\overrightarrow{a}}_{3}={\overrightarrow{a}}_{2}-{\overrightarrow{a}}_{1}$ are the lattice vectors. Diagonalizing the Hamiltonian gives three magnon branches of the kagomé ferromagnet. In the following we set *B* = 0 as it simply shifts the magnon bands to high energy. As shown in Fig. (1)c, without the DM interaction, i.e. *D*/*J* = 0 or *φ* = 0, the two lower dispersive bands form Dirac magnon cones at ±**K** (see Fig. (1)b), whereas the flat band has the highest energy and touches one of the dispersive bands quadratically at Γ. In Fig. (1)d, we include a small DM interaction *D*/*J* = 0.15 applicable to Cu(1–3, bdc)^16^. Now the flat band acquires a dispersion and all the bands are separated by a finite energy gap with well-defined Chern numbers. Thus, the system becomes a topological magnon insulator^7,8,16,17^.

### Periodically driven topological magnon insulators

In this section, we introduce the notion of periodically driven intrinsic topological magnon insulators. Essentially, this concept will be based on the charge-neutrality of magnons in combination with their magnetic dipole moment $\overrightarrow{\mu }=\mu \hat{z}$ with $\mu =g{\mu }_{B}$. Let us suppose that magnons in insulating quantum magnetic systems are exposed to an electromagnetic field with a dominant time-dependent electric field vector $\overrightarrow{E}(\tau )$. Then the effects of the field on the system can be described by a vector potential defined as $\overrightarrow{E}(\tau )=-\partial \overrightarrow{A}(\tau )/\partial \tau$, where $\overrightarrow{A}(\tau )=[{A}_{x}\,\sin (\omega \tau ),{A}_{y}\,\sin (\omega \tau +\varphi ),0]$ with amplitudes *A*_*x*_ and *A*_*y*_, frequency *ω*, and phase difference *ϕ*. The vector potential has time-periodicity: $\overrightarrow{A}(\tau +T)=\overrightarrow{A}(\tau )$, with $T=2\pi /\omega$ being the period. Here *ϕ* = *π*/2 corresponds to circularly-polarized light, whereas *ϕ* = (0, *π*) corresponds to linearly-polarized light.

Using the AC effect for charge-neutral particles^72^, we consider magnon quasiparticles with magnetic dipole *μ* moving in the background of a time-dependent electric field. In this scenario they will acquire a time-dependent AC phase (see Methods) given by

4 $${A}_{\ell {\ell }^{\text{'}}}(\tau )=\mu {\int }_{{\overrightarrow{r}}_{\ell }}^{{\overrightarrow{r}}_{{\ell }^{\text{'}}}}\overrightarrow{A}(\tau )\cdot d\overrightarrow{\ell },$$

where $\hslash =c=1$ has been used, and ${\overrightarrow{r}}_{\ell }$ is the coordinate of the lattice at site $\ell$. By virtue of the time-dependent Peierls substitution, the periodically driven magnon Hamiltonian is succinctly given by

5 $$ {\mathcal H} (\tau )=-{t}_{0}\sum _{\langle \ell \ell ^{\prime} \rangle }({e}^{i[{\phi }_{\ell \ell ^{\prime} }+{A}_{\ell \ell ^{\prime} }(\tau )]}{a}_{\ell }^{\dagger }{a}_{\ell ^{\prime} }+{\rm{H}}{\rm{.c}})+{t}_{z}\sum _{\ell }{n}_{\ell }.$$

Therefore, the time-dependent momentum space Hamiltonian ${\mathcal H} (\overrightarrow{k},\tau )$ corresponds to making the time-dependent Peierls substitution $\overrightarrow{k}\to \overrightarrow{k}+\overrightarrow{A}(\tau )$ in Eq. (3). We note that previous studies based on the AC effect in insulating magnets considered a time-independent electric field gradient, which leads to magnonic Landau levels^67–70^. In stark contrast to those studies, the time-dependent version can lead to Floquet topological magnon insulators in insulating quantum magnets with inversion symmetry, e.g. the honeycomb lattice^71^ or the Lieb lattice (see Supplementary Information). We note that Floquet topological magnon insulators can also be generated by driving a gapped trivial magnon insulator with vanishing Chern number, in a similar manner to Dirac magnons. A comprehensive study of this case is beyond the purview of this report. In the current study, however, the kagomé lattice quantum ferromagnets naturally lack inversion symmetry, and thus allows an intrinsic DM interaction as depicted in Fig. (1)a.

Now, we apply the magnonic Floquet-Bloch theory in Methods. For simplicity, we consider the magnonic Floquet Hamiltonian in the off-resonant regime, when the driving frequency *ω* is larger than the magnon bandwidth Δ of the undriven system, i.e. ω $\gg$ Δ. In this limit, the Floquet bands are decoupled, and it suffices to consider the zeroth order time-independent Floquet magnon Hamiltonian ${ {\mathcal H} }^{0}(\overrightarrow{k})={t}_{z}{{\rm{I}}}_{3\times 3}-{{\rm{\Lambda }}}^{0}(\overrightarrow{k})$, where

6 $${{\rm{\Lambda }}}^{0}(\overrightarrow{k})=(\begin{array}{ccc}0 & {t}_{0}^{AB}\,\cos \,{k}_{2}{e}^{-i\phi } & {t}_{0}^{CA}\,\cos \,{k}_{3}{e}^{i\phi }\\ {t}_{0}^{AB}\,\cos \,{k}_{2}{e}^{i\phi } & 0 & {t}_{0}^{BC}\,\cos \,{k}_{1}{e}^{-i\phi }\\ {t}_{0}^{CA}\,\cos \,{k}_{3}{e}^{-i\phi } & {t}_{0}^{BC}\,\cos \,{k}_{1}{e}^{i\phi } & 0\end{array}),$$

and

7 $${t}_{0}^{AB}=2{t}_{0}{{\mathscr{J}}}_{0}(\frac{1}{2}\sqrt{{A}_{x}^{2}+3{A}_{y}^{2}+2\sqrt{3}{A}_{x}{A}_{y}\,\cos \,\varphi }),$$

8 $${t}_{0}^{BC}=2{t}_{0}{{\mathscr{J}}}_{0}(|{A}_{x}|),$$

9 $${t}_{0}^{CA}\mathrm{=2}{t}_{0}{{\mathscr{J}}}_{0}(\frac{1}{2}\sqrt{{A}_{x}^{2}+3{A}_{y}^{2}-2\sqrt{3}{A}_{x}{A}_{y}\,\cos \,\varphi }),$$

where ${{\mathscr{J}}}_{n}(x)$ is the Bessel function of order *n*. Evidently, a direct consequence of photo-irradiation is that the magnonic Floquet Hamiltonian (6) is equivalent to that of a distorted kagomé ferromagnet with unequal tunable interactions ${t}_{0}^{AB}\ne {t}_{0}^{BC}\ne {t}_{0}^{CA}$. In the following, we shall discuss the topological aspects of this model. The Berry curvature is one of the main important quantities in topological systems. It is the basis of many observables in topological insulators. To study the photoinduced topological phase transitions in driven topological magnon insulators, we define the Berry curvature of a given magnon band *α* as

10 $${{\rm{\Omega }}}_{\alpha }(\overrightarrow{k})=-\sum _{\alpha ^{\prime} \ne \alpha }\frac{2{\rm{Im}}(\langle {\psi }_{\overrightarrow{k},\alpha }|{\hat{v}}_{x}|{\psi }_{\overrightarrow{k},\alpha ^{\prime} }\rangle \langle {\psi }_{\overrightarrow{k},\alpha ^{\prime} }|{\hat{v}}_{y}|{\psi }_{\overrightarrow{k},\alpha }\rangle )}{{({\epsilon }_{\overrightarrow{k},\alpha }-{\epsilon }_{\overrightarrow{k},\alpha ^{\prime} })}^{2}},$$

where ${\hat{v}}_{x,y}=\partial { {\mathcal H} }^{0}(\overrightarrow{k})/\partial {k}_{x,y}$ are the velocity operators, ${\psi }_{\overrightarrow{k},\alpha }$ are the magnon eigenvectors, and ${\epsilon }_{\overrightarrow{k},\alpha }$ are the magnon energy bands. The associated Chern number is defined as the integration of the Berry curvature over the Brillouin zone (BZ),

11 $${C}_{\alpha }=\frac{1}{2\pi }{\int }_{BZ}{d}^{2}k{{\rm{\Omega }}}_{\alpha }(\overrightarrow{k}),$$

where *α* = 1, 2, 3 label the lower, middle, and upper magnon bands respectively.

**Figure 2:**
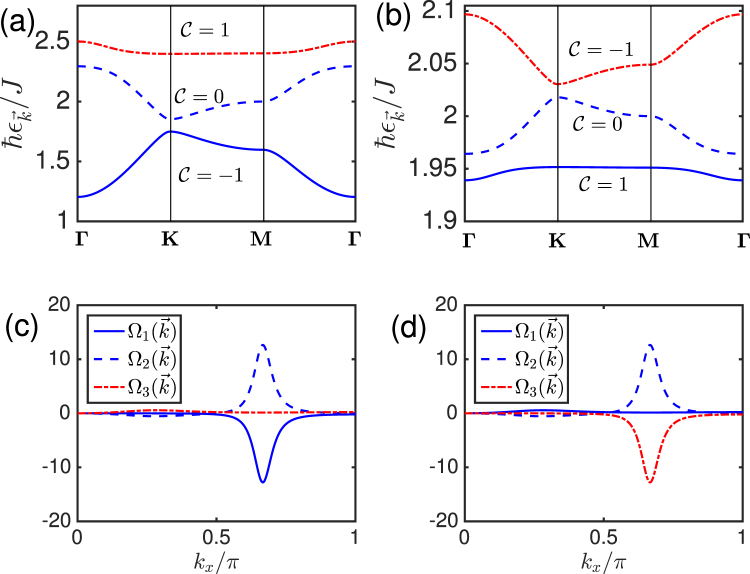
Top panel. Topological magnon bands of periodically driven topological magnon insulator at *D*/*J* = 0.15. (**a**) *A*_*x*_ = *A*_*y*_ = 1.7 and *ϕ* = *π*/2. (**b**) *A*_*x*_ = *A*_*y*_ = 2.5 and *ϕ* = *π*/2. Bottom panel. Tunable Berry curvatures of periodically driven intrinsic topological magnon insulator on the kagomé lattice at *k*_*y*_ = 0 and *D*/*J* = 0.15. (**c**) ${A}_{x}={A}_{y}=1.7$ and *ϕ* = *π*/2. (**d**) ${A}_{x}={A}_{y}=2.5$ and *ϕ* = *π*/2.

**Figure 3:**
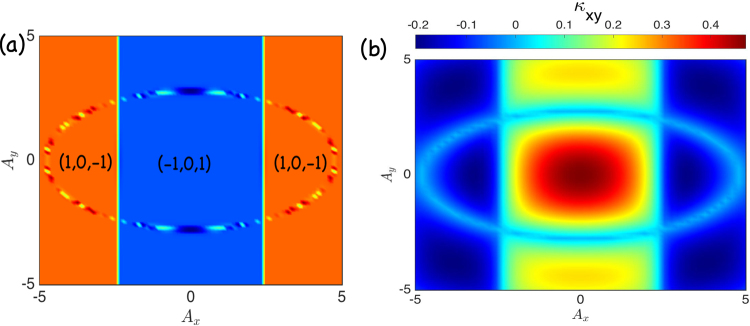
Topological phase diagram of periodically driven intrinsic topological magnon insulator on the kagomé lattice. (**a**) Chern number phase diagram for $D/J=0.15$ and *ϕ* = *π*/2. (**b**) Thermal Hall conductivity phase diagram for $D/J=0.15$, *ϕ* = *π*/2, and $T=0.75$.

In Fig. 2 we have shown the evolution of the magnon bands and the Berry curvatures for varying light intensity. We can see that the lower and upper magnon bands and their corresponding Berry curvatures change with varying light intensity, whereas the middle magnon band remains unchanged. Consequently, the system changes from one topological magnon insulator with Chern numbers (−1, 0, 1) to another one with Chern numbers (1, 0, −1) as shown in the photoinduced topological phase diagram in Fig. 3(a). In other words, exposing a topological magnon insulator to a varying light intensity field redistribute the magnon band structures and subsequently leads to a topological phase transition from one topological magnon insulator to another with different Berry curvatures and Chern numbers.

A crucial consequence of topological magnon insulators is the thermal Hall effect^74,75^. Theoretically, the thermal Hall effect is understood as a consequence of the Berry curvatures induced by the DM interaction^6,76,77^. If we focus on the regime in which the Bose distribution function is close to equilibrium, the same theoretical concept of undriven thermal Hall effect can be applied to the photoinduced system. The transverse component *κ*_*xy*_ of the thermal Hall conductivity is given explicitly as^76,77^

12 $${\kappa }_{xy}=-{k}_{B}^{2}T{\int }_{BZ}\frac{{d}^{2}k}{{\mathrm{(2}\pi )}^{2}}\sum _{\alpha =1}^{N}{c}_{2}({n}_{\alpha }){{\rm{\Omega }}}_{\alpha }(\overrightarrow{k}),$$

where ${n}_{\alpha }=n[{\epsilon }_{\alpha }(\overrightarrow{k})]=\mathrm{1/}[{e}^{{\epsilon }_{\alpha }(\overrightarrow{k})/{k}_{B}T}-1]$ is the Bose distribution function close to thermal equilibrium, *k*_*B*_ is the Boltzmann constant, *T* is the temperature, and ${c}_{2}(x)=\mathrm{(1}+x){(\mathrm{ln}\frac{1+x}{x})}^{2}-{(\mathrm{ln}x)}^{2}-2{{\rm{Li}}}_{2}(-x)$, with ${{\rm{Li}}}_{2}(x)$ being the dilogarithm. Indeed, the thermal Hall conductivity is the Berry curvature weighed by the *c*_2_ function. Therefore, any change in the Berry curvature will affect the thermal Hall conductivity. Evidently, as shown in Fig. 3(b), the two photoinduced phases in the intrinsic topological magnon insulator have different signs of the anomalous thermal Hall conductivity due to the sign change in the Berry curvatures. The elliptic ring in the topological phase diagram in Fig. 3 is an artifact of the kagomé lattice, together with circularly polarized light. It does not exist with linearly polarized light, and it is also not present on the honeycomb lattice.

### Photoinduced topological magnon semimetal

The topological phase transitions in periodically driven intrinsic topological magnon insulators can also be extended to synthetic topological magnon semimetals with gapless magnon bands. As we mentioned above the photoinduced distorted interactions ${t}_{0}^{AB}\ne {t}_{0}^{BC}\ne {t}_{0}^{CA}$ can be controlled by the amplitude and the polarization of the light intensity, therefore there is a possibility to obtain new interesting magnon phases in periodically driven intrinsic topological magnon insulators. Let us consider three different limiting cases of the photoinduced distorted interactions.

(i): ${t}_{0}^{AB}=0;\,{t}_{0}^{BC}\ne {t}_{0}^{CA}\ne 0$, which leads to the magnon bands ${\epsilon }_{\overrightarrow{k}}^{0}={t}_{z}$ and

13 $${\epsilon }_{\overrightarrow{k}}^{\pm }={t}_{z}\pm \frac{1}{\sqrt{2}}\sqrt{{({t}_{0}^{BC})}^{2}\mathrm{[1}+\,\cos \,\mathrm{(2}{k}_{1})]+{({t}_{0}^{CA})}^{2}\mathrm{[1}+\,\cos \,\mathrm{(2}{k}_{3})]}$$

(ii): ${t}_{0}^{BC}=0;{t}_{0}^{AB}\ne {t}_{0}^{CA}\ne 0$. The magnon bands in this case are given by ${\epsilon }_{\overrightarrow{k}}^{0}={t}_{z}$ and

14 $${\epsilon }_{\overrightarrow{k}}^{\pm }={t}_{z}\pm \frac{1}{\sqrt{2}}\sqrt{{({t}_{0}^{AB})}^{2}\mathrm{[1}+\,\cos \,\mathrm{(2}{k}_{2})]+{({t}_{0}^{CA})}^{2}\mathrm{[1}+\,\cos \,\mathrm{(2}{k}_{3})]}$$

(iii): ${t}_{0}^{CA}=0;\,{t}_{0}^{BC}\ne {t}_{0}^{CA}\ne 0$. In this case we have ${\epsilon }_{\overrightarrow{k}}^{0}={t}_{z}$ and

15 $${\epsilon }_{\overrightarrow{k}}^{\pm }={t}_{z}\pm \frac{1}{\sqrt{2}}\sqrt{{({t}_{0}^{AB})}^{2}\mathrm{[1}+\,\cos \,\mathrm{(2}{k}_{2})]+{({t}_{0}^{BC})}^{2}\mathrm{[1}+\,\cos \,\mathrm{(2}{k}_{1})]}$$

**Figure 4:**
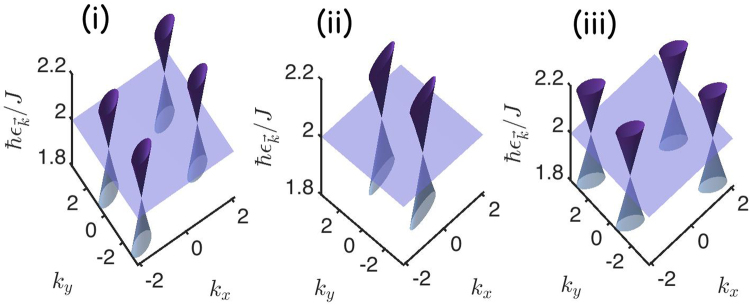
Photoinduced pseudospin-1 topological magnon semimetals in periodically driven intrinsic topological magnon insulator on the kagomé lattice. (i) ${t}_{0}^{AB}=\mathrm{0;}\,{t}_{0}^{BC}\ne {t}_{0}^{CA}\ne 0$ with ${A}_{x}=1.7$, ${A}_{y}=1.5$ and *ϕ* = *π*/2. (ii) ${t}_{0}^{BC}=\mathrm{0;}\,{t}_{0}^{AB}\ne {t}_{0}^{CA}\ne 0$ with *A*_*x*_ = 1.7, *A*_*y*_ = 2.5 and *ϕ* = 0. (iii) ${t}_{0}^{CA}=0;\,{t}_{0}^{BC}\ne {t}_{0}^{CA}\ne 0$ with *A*_*x*_ = 1.7, *A*_*y*_ = 1.5 and *ϕ* = *π*/2. Here we set *D*/*J* = 0.15.

In each case there are three magnon bands featuring one flat magnon band and two dispersive magnon bands, similar to the undriven topological magnon insulator in Fig. (1)d. However, in the present case there is a possibility to obtain other interesting magnon phases different from the gapped topological magnon bands in the undriven system. For instance, cases (i)–(iii) realize pseudospin-1 Dirac-Weyl magnon cones or three-component bosons at ${{\bf{K}}}_{1}=(\pm \pi \mathrm{/2},\mp \pi \mathrm{/2}\sqrt{3})$, ${{\bf{K}}}_{2}=(0,\mp \pi /\sqrt{3})$, and ${{\bf{K}}}_{3}=(\pm \pi \mathrm{/2},\pm \pi \mathrm{/2}\sqrt{3})$ respectively, as indicated by red and green dots in Fig. 1(b). The pseudospin-1 Dirac-Weyl magnon cones occur at the energy of the flat band ${\epsilon }_{{{\bf{K}}}_{i}}={t}_{z}$ as shown in Fig. (4). Expanding the Floquet-Bloch magnon Hamiltonian in the vicinity of **K**_1_ yields

16 $${ {\mathcal H} }^{0}({{\bf{K}}}_{1}+\overrightarrow{q})\simeq {t}_{z}{{\rm{I}}}_{3\times 3}\pm {v}_{x}{q}_{x}{\lambda }_{x}\mp {v}_{y}{q}_{y}{\lambda }_{y},$$

where ${v}_{x}={t}_{0}^{BC}$ and ${v}_{y}={t}_{0}^{CA}$. The *λ*’*s* are the pseudospin-1 representation of the SU(2) Lie algebra $[{\lambda }_{i},{\lambda }_{j}]=i{\epsilon }_{ijk}{\lambda }_{k}$, where

17 $${\lambda }_{x}=(\begin{array}{ccc}0 & 0 & {e}^{i\phi }\\ 0 & 0 & 0\\ {e}^{-i\phi } & 0 & 0\end{array}),\,{\lambda }_{y}=(\begin{array}{ccc}0 & 0 & 0\\ 0 & 0 & {e}^{-i\phi }\\ 0 & {e}^{i\phi } & 0\end{array}),\,{\lambda }_{z}=(\begin{array}{ccc}0 & i{e}^{2i\phi } & 0\\ -i{e}^{-2i\phi } & 0 & 0\\ 0 & 0 & 0\end{array}).$$

Similar pseudospin-1 linear Hamiltonian can be obtained for the Dirac-Weyl magnon cones around **K**_2_ and **K**_3_.

## Conclusion

We have presented a study of photoinduced topological phase transitions in periodically driven intrinsic topological magnon insulators. The main result of this report is that intrinsic topological magnon insulators in the kagomé ferromagnets can be driven to different topological phases with different Berry curvatures using photo-irradiation. Therefore, each topological phase is associated with a different sign of the thermal Hall conductivity, which results in a sign reversal of the magnon heat photocurrent. These topological transitions require no external magnetic field. Interestingly, we observed that by varying the light intensity, the periodically driven intrinsic topological magnon insulators can also realize synthetic gapless topological magnon semimetals with pseudospin-1 Dirac-Weyl magnon cones. We believe that our results should also apply to 3D topological magnon insulators. In fact, a 3D topological magnon insulator should also have a Dirac magnon cone on its 2D surface, which can be photo-irradiated to engineer a 2D topological magnon insulator in analogy to electronic systems^44^. Here, we have studied the off-resonant regime, when the driving frequency *ω* is larger than the magnon bandwidth Δ of the undriven system. In this regime, the Floquet sidebands are decoupled and can be considered independently. By lowering the driving frequency below the magnon bandwidth, the Floquet sidebands overlap, which results in photon absorption. In this limit the system would have several overlapping topological phases depending on the polarization of the light. In general, we believe that the predicted results in this report are pertinent to experiments and will remarkably impact future research in topological magnon insulators and their potential practical applications to photo-magnonics^30^ and magnon spintronics^31,32^.

## Methods

### Neutral particle with magnetic dipole moment in an external electromagnetic field

Two-dimensional topological magnon insulators (or Dirac magnons) can be captured by massive (or massless) (2 + 1)-dimensional Dirac equation near ±**K**. In general, a massive neutral particle with mass (*m*) couples non-minimally to an external electromagnetic field (denoted by the tensor *F*_*μv*_) via its magnetic dipole moment (*μ*). In (3 + 1) dimensions, the system is described by the Dirac-Pauli Lagrangian^78^

18 $$ {\mathcal L} =\bar{\psi }(x)(i{\gamma }^{\mu }{\partial }_{\mu }-\frac{\mu }{2}{\sigma }^{\mu \nu }{F}_{\mu \nu }-m)\psi (x),$$

where $\hslash =c=1$ has been used. Here $x\equiv {x}^{\mu }=({x}^{0},\overrightarrow{x})$, $\bar{\psi }(x)={\psi }^{\dagger }(x){\gamma }^{0}$, and ${\gamma }^{\mu }=({\gamma }^{0},\overrightarrow{\gamma })$ are the 4 × 4 Dirac matrices that obey the algebra

19 $$\{{\gamma }^{\mu },{\gamma }^{\nu }\}=2{g}^{\mu \nu },\,{\rm{where}}\quad {g}^{\mu \nu }={\rm{diag}}\mathrm{(1},-1,-1,-1),$$

and

20 $${\sigma }^{\mu \nu }=\frac{i}{2}[{\gamma }^{\mu },{\gamma }^{\nu }]=i{\gamma }^{\mu }{\gamma }^{\nu },\quad (\mu \ne \nu \mathrm{)}.$$

For the purpose of our study in this report, we consider an electromagnetic field with only spatially uniform and time-varying electric field vector $\overrightarrow{E}(\tau )$ (however, the resulting AC phase is valid for a general electric field $\overrightarrow{E}(\tau ,\overrightarrow{r})$). In this case, the corresponding Hamiltonian is given by

21 $$ {\mathcal H} =\int {d}^{3}x\,{\psi }^{\dagger }(x)[\overrightarrow{\alpha }\cdot (-i\overrightarrow{\nabla }-i\mu \beta \overrightarrow{E}(\tau ))+m\beta ]\psi (x),$$

where $$\overrightarrow{\alpha }={\gamma }^{0}\overrightarrow{\gamma }$$, and $$\beta ={\gamma }^{0}$$.

In (2 + 1) dimensions, the Hamiltonian (21) corresponds to that of 2D topological magnon insulators (near ±**K**) with magnetic dipole moment $\overrightarrow{\mu }=\mu \hat{z}$, where *μ* = *gμ*_*B*_. In this case, the Dirac matrices are simply Pauli matrices given by

22 $$\beta ={\gamma }^{0}={\sigma }_{z},\,{\gamma }^{1}=i{\sigma }_{y},\,{\gamma }^{2}=-i{\sigma }_{x}.$$

The corresponding momentum space Hamiltonian in (2 + 1) dimensions now takes the form

23 $$ {\mathcal H} =\int \frac{{d}^{2}k}{{\mathrm{(2}\pi )}^{2}}\,{\psi }^{\dagger }(\overrightarrow{k},\tau ) {\mathcal H} (\overrightarrow{k},\tau )\psi (\overrightarrow{k},\tau ),$$

where

24 $$ {\mathcal H} (\overrightarrow{k},\tau )=\overrightarrow{\sigma }\cdot [\overrightarrow{k}+\mu (\overrightarrow{E}(\tau )\times \hat{z})]+m{\sigma }_{z},\,{\rm{with}}\,\overrightarrow{\sigma }=({\sigma }_{x},{\sigma }_{y}\mathrm{)}.$$

We can clearly see the time-dependent AC phase from the Hamiltonian in Eq. 24. Since $\overrightarrow{E}(\tau )=-\partial \overrightarrow{A}(\tau )/\partial \tau$, we can replace $\overrightarrow{E}(\tau )\times \hat{z}$ with $\overrightarrow{A}(\tau )$ as in Eq. (4). We note that this replacement does not change our results, because we could also define the time-periodic electric field $\overrightarrow{E}(\tau )$ such that $\overrightarrow{E}(\tau )\times \hat{z}=[{E}_{x}\,\sin (\omega \tau ),{E}_{y}\,\sin (\omega \tau +\varphi \mathrm{),0]}$, where *E*_*x*,*y*_ is now equivalent to *A*_*x*,*y*_ in Eq. (4).

### Magnonic Floquet-Bloch theory

Periodically driven quantum systems are best described by the Floquet-Bloch theory. The magnonic version describes the interaction of light with magnonic Bloch states in insulating magnets. In this section, we develop this theory for the time-dependent magnon Hamiltonian Eq. (5) in momentum space. We consider the time-dependent Schrödinger equation for the system

25 $$i\hslash \frac{d|\psi (\overrightarrow{k},\tau )\rangle }{d\tau }= {\mathcal H} (\overrightarrow{k},\tau )|\psi (\overrightarrow{k},\tau )\rangle ,$$

where |$\psi (\overrightarrow{k},\tau )$ is the driven wave function. Due to the periodicity of the vector potential $\overrightarrow{A}(\tau )$, the driven Hamiltonian ${\mathcal H} (\overrightarrow{k},\tau )$ is also periodic and can be expanded in Fourier space as

26 $$ {\mathcal H} (\overrightarrow{k},\tau )= {\mathcal H} (\overrightarrow{k},\tau +T)=\sum _{n=-\infty }^{\infty }{e}^{in\omega \tau }{ {\mathcal H} }_{n}(\overrightarrow{k}),$$

where ${ {\mathcal H} }_{n}(\overrightarrow{k})=\frac{1}{T}{\int }_{0}^{T}{e}^{-in\omega \tau } {\mathcal H} (\overrightarrow{k},\tau )d\tau ={ {\mathcal H} }_{-n}^{\dagger }(\overrightarrow{k})$ is the Fourier component. The ansatz for solution to the Schrödinger equation can be written as

27 $$|{\psi }_{\alpha }(\overrightarrow{k},\tau )\rangle ={e}^{-i{\epsilon }_{\alpha }(\overrightarrow{k})\tau }|{\xi }_{\alpha }(\overrightarrow{k},\tau )\rangle ={e}^{-i{\epsilon }_{\alpha }(\overrightarrow{k})\tau }\sum _{n=-\infty }^{\infty }{e}^{in\omega \tau }|{\xi }_{\alpha },n(\overrightarrow{k})\rangle $$

where |${\xi }_{\alpha }(\overrightarrow{k},\tau )$〉 is the time-periodic Floquet-Bloch wave function of magnons and ${\epsilon }_{\alpha }(\overrightarrow{k})$ are the magnon quasi-energies. The corresponding Floquet-Bloch eigenvalue equation is given by ${ {\mathcal H} }_{F}(\overrightarrow{k},\tau )|{\xi }_{\alpha }(\overrightarrow{k},\tau )\rangle =$ ${\epsilon }_{\alpha }(\overrightarrow{k})|{\xi }_{\alpha }(\overrightarrow{k},\tau )\rangle$, where ${ {\mathcal H} }_{F}(\overrightarrow{k},\tau )= {\mathcal H} (\overrightarrow{k},\tau )-i{\partial }_{\tau }$ is the Floquet operator. This leads to a time-independent Floquet eigenvalue equation

28 $$\sum _{m}[{ {\mathcal H} }^{n-m}(\overrightarrow{k})+m\omega {\delta }_{n,m}]{\xi }_{\alpha }^{m}(\overrightarrow{k})={\epsilon }_{\alpha }(\overrightarrow{k}){\xi }_{\alpha }^{n}(\overrightarrow{k}),$$

where ${ {\mathcal H} }^{p}(\overrightarrow{k})=\frac{1}{T}{\int }_{0}^{T}d\tau {e}^{-ip\omega \tau }{ {\mathcal H} }^{p}(\overrightarrow{k},\tau )$. The associated Bessel function integral is given by

29 $$\frac{1}{T}\,{\int }_{0}^{T}\,d\tau {e}^{-ip\omega \tau }{e}^{iz\sin (\omega \tau )}{e}^{iz^{\prime} \sin (\omega \tau +\varphi )}={e}^{ip\arctan (\frac{z^{\prime} \sin (\varphi )}{z+z^{\prime} \cos (\varphi )})}{{\mathscr{J}}}_{p}(\sqrt{{z}^{2}+z{^{\prime} }^{2}+2zz^{\prime} \,\cos (\varphi )}),$$

where ${{\mathscr{J}}}_{p}$ is the Bessel function of order *p*. In Eq. (6) we consider the zeroth order approximation corresponding to *p* = 0.

## Electronic supplementary material

Supplementary Information
