# Supplementary material for: Photoinduced Topological Phase Transitions in Topological Magnon Insulators
Source: Sci Rep. 2018 Mar 13;8:4431. doi: 10.1038/s41598-018-22779-8 (PMC5849775; doi:10.1038/s41598-018-22779-8)
Supplement: Supplementary file 1 — Supplementary Information [file 41598_2018_22779_MOESM1_ESM.pdf]

Photoinduced Topological Phase Transitions in Topological Magnon Insulators Supplementary Information

S. A. Owerre[1]

[1] *Perimeter Institute for Theoretical Physics, 31 Caroline St. N., Waterloo, Ontario N2L 2Y5, Canada.*

(Dated: December 16, 2017)

Lieb lattice ferromagnets. Although topological magnon insulators can exist in different lattice geometries [5–13], their experimental observation in real magnetic materials has been elusive [14] due to some of the following factors:

1. In realistic physical magnetic materials, the DM interaction which is necessary for topological magnon insulators to exist can be very weak or even negligible.

2. Magnon excitations in realistic physical magnetic materials are not always clearly resolved in the inelastic neutron scattering experiments. Therefore distinctive magnon bands might not be very visible in the experimental data.

3. In many cases, realistic physical magnetic materials may have inversion symmetry which forbids an intrinsic DM interaction.

Unlike kagomé ferromagnets, the Lieb lattice insulating ferromagnets fall within the third factor. They can also be considered as the 2D version of the perovskite lattice for which magnon Hall measurement (which is due to topological magnon bands) shows vanishing behaviour in some materials [15], possibly due to cubic or inversion symmetry and/or very weak DM interaction. They also exist in the planes of weakly-coupled cuprate superconductors [16, 17]. The magnon bands of the 2D Lieb lattice have two dispersive bands and one flat band, and they linearly touch at isolated point in the Brillouin zone and form a pseudospin-1 Dirac magnon point. The flat band on the Lieb lattice has been the focus of many theoretical and experimental studies in optical lattices [18–23].

In the following, we apply the approach in the previous work [24] to insulating ferromagnets on the Lieb lattice. The goal is to induce a synthetic DM interaction on the Lieb lattice, and subsequently study its topological features.

Pseudospin-1 Dirac magnon on the Lieb lattice. First, we consider the Heisenberg ferromagnetic spin model on a 2D Lieb lattice

$$\mathcal{H} = -J\sum_{\langle \ell\ell'\rangle}\vec{S}_\ell\cdot\vec{S}_{\ell'} - B\sum_\ell S_\ell^z, \tag{1}$$

where ℓ and ℓ' denote the sites on the Lieb lattice and the first summation is taken over the nearest-neighbour (NN) sites. The second term is an external magnetic field applied along the z direction with $B = g\mu_B H$ where μ_B is the Bohr magneton, g the spin g-factor, and H is the strength of the magnetic field. As we are interested in the underlying magnetic excitations of Eq. (1), it is advantageous to introduce the Holstein-Primakoff transformation: $S_\ell^z = S - a_\ell^\dagger a_\ell,\ S_\ell^+ \approx \sqrt{2S}a_\ell = (S_\ell^-)^\dagger$, where $a_\ell^\dagger(a_\ell)$ are the bosonic creation (annihilation) operators, and $S_\ell^\pm = S_\ell^x \pm iS_\ell^y$ denote the spin creation and annihilation operators which correspond to the hopping terms. The corresponding noninteracting magnon Hamiltonian is given by

$$\mathcal{H} = -t_0\sum_{\langle \ell\ell'\rangle}\left(a_\ell^\dagger a_{\ell'} + \text{H.c}\right) + t_z\sum_\ell n_\ell, \tag{2}$$

where $n_\ell = a_\ell^\dagger a_\ell$ is the number operator, $t_0 = JS$ and $t_z = 4JS + B$ with $t_z > t_0$. In Fourier space the Hamiltonian is given by $\mathcal{H} = \sum_{\vec{k}}\psi_{\vec{k}}^\dagger\mathcal{H}(\vec{k})\psi_{\vec{k}}$, with $\psi_{\vec{k}} = (a_{\vec{k},1}, a_{\vec{k},2}, a_{\vec{k},3})^{\rm T}$, where

$$\mathcal{H}(\vec{k}) = \begin{pmatrix} t_z & -2t_0\cos(k_x) & -2t_0\cos(k_y)\\ -2t_0\cos(k_x) & t_z & 0\\ -2t_0\cos(k_y) & 0 & t_z\end{pmatrix}, \tag{3}$$

with $k_{x,y} \in (-\pi, \pi)$ in the first Brillouin zone. The eigenvalues of Eq. (3) are given by $\epsilon_0(\vec{k}) = t_z$ and

$$\epsilon_\pm(\vec{k}) = t_z \pm t_0\sqrt{4 + 2\cos(2k_x) + 2\cos(2k_y)}. \tag{4}$$

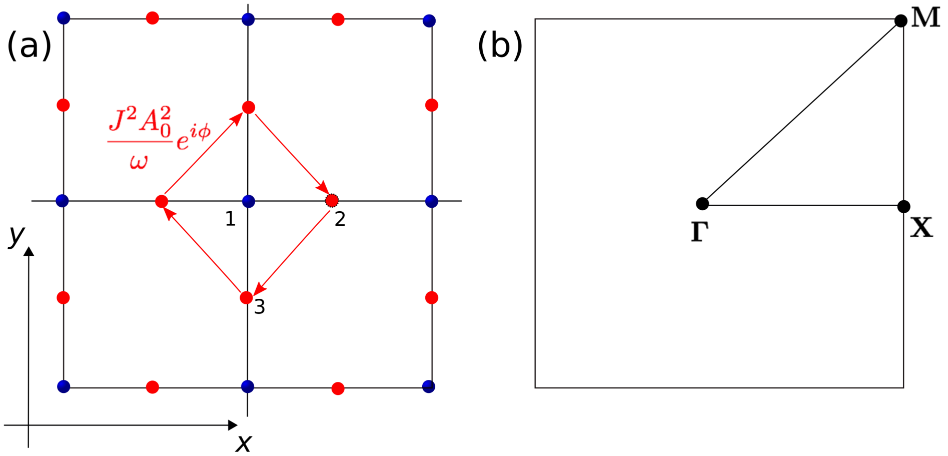

FIG. 1: (a) Schematic of the Lieb lattice. The unit cell consists of three sites labeled by numbers, and the arrows indicate laser-induced next-nearest neighbour interactions. (b) The corresponding Brillouin zone of the lattice with indicated paths.

The three magnon bands touch linearly at the **M** point ($k_x = k_y = \pi/2$) in the Brillouin zone (see Fig. (1)) and form a pseudospin-1 Dirac magnon cone or three-component boson beyond that of honeycomb lattice [25].

Periodically driven pseudospin-1 Dirac magnon on the Lieb lattice. As we mentioned in the main text, the best way to construct Floquet topological magnons, e.g. on the honeycomb lattice [24] or magnonic Landau levels [26] is to take advantage of the magnetic dipole moment carried by magnons, i.e. $\vec{\mu} = g\mu_B\hat{z}$. Following the procedure in the main text, we obtain the periodically driven magnon Hamiltonian

$$\mathcal{H}(t) = -t_0\sum_{\langle \ell\ell'\rangle}\left(e^{iA_{\ell\ell'}(\tau,\phi)}a_\ell^\dagger a_{\ell'} + \text{H.c}\right) + t_z\sum_\ell n_\ell. \tag{5}$$

The main observation in Eq. (5) is that an uncharged particle with a magnetic dipole moment μ_z couples to an electromagnetic vector potential via the Aharonov-Casher phase in the same way a charged particle couples via the Aharonov-Bohm phase. In momentum space, the time-periodic Hamiltonian $\mathcal{H}(\vec{k},\tau,\phi)$ corresponds to making the transformation $\vec{k} \to \vec{k} + \vec{A}(\tau,\phi)$ in Eq. (3).

Floquet magnon Chern insulator on the Lieb lattice. The Floquet formalism enables us to transform the periodically driven magnon Hamiltonian in Eq. (5) to a static one. This can be done by considering the high frequency limit $\hbar\omega \gg J$ in our case. The corresponding effective Floquet-Bloch magnon Hamiltonian is given by

$$\mathcal{H}_{\text{eff}}(\vec{k},\phi) = \mathcal{H}_0(\vec{k},\phi) + \sum_{n\geq 1}\frac{\left[\mathcal{H}_n(\vec{k},\phi), \mathcal{H}_{-n}(\vec{k},\phi)\right]}{n\omega} + \mathcal{O}\left(\frac{1}{\omega^2}\right), \tag{6}$$

where the last term denotes a commutation relation. We start from the time-dependent momentum space Hamiltonian $\mathcal{H}(\vec{k},\tau,\phi)$ as given by Eq. (3) with $\vec{k} = \vec{k} + \vec{A}(\tau,\phi)$. Next, we use the standard relation

$$\exp[iz\sin(x)] = \sum_{n=-\infty}^{\infty}\mathcal{J}_n(z)e^{inx}, \tag{7}$$

where $\mathcal{J}_n(z)$ is the Bessel function of order $n \in \mathbb{Z}$ and $\mathcal{J}_{-n}(z) = (-)^n\mathcal{J}_n(z)$. We can approximate the periodically driven magnon Hamiltonian as $\mathcal{H}(\vec{k},\tau,\phi) \approx \mathcal{H}_0(\vec{k},\phi) + \mathcal{H}_1(\vec{k},\phi)e^{i\omega\tau} + \mathcal{H}_{-1}(\vec{k},\phi)e^{-i\omega\tau}$ and assume that higher order terms with $|n| \geq 2$ are negligible. The zeroth order Fourier coefficient of the time-dependent magnon Hamiltonian is given by $\mathcal{H}_0(\vec{k},\phi)$ and it is the same as $\mathcal{H}(\vec{k},\phi)$ in Eq. (3) with $t_0 = \Delta_0$, where $\Delta_0 = t_0\mathcal{J}_0(A_0)$ with $A_0 = A_x = A_y$. Therefore the zeroth order term simply renormalizes the NN interaction with no breaking of time-reversal symmetry. The first order term is given by

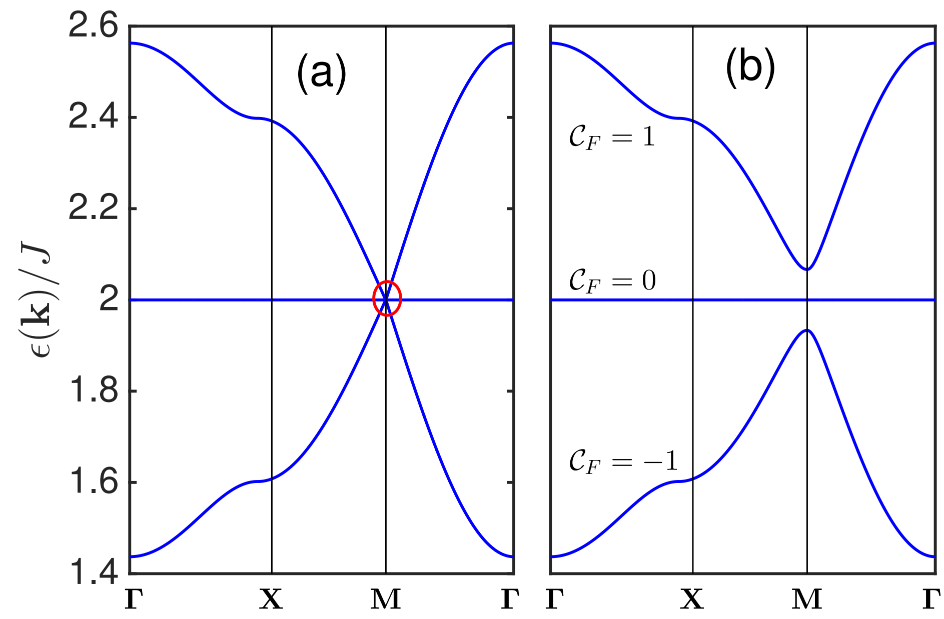

FIG. 2: (a) Floquet Dirac magnon bands with pseudospin-1 Dirac point (red circle) in the zeroth order approximation (i.e $\Delta_1 = 0$). (b) Floquet topological magnon bands and the corresponding Chern numbers in the first order approximation (i.e $\Delta_1 \neq 0$) with the parameters set to $A_0 = 1.7$, $\hbar\omega = 10J$, $\phi = \pi/2$, $B = 0$, and $S = 1/2$.

$$\mathcal{H}_1(\vec{k},\phi) = i\rho \begin{pmatrix} 0 & \sin(k_x) & e^{-i\phi}\sin(k_y) \\ \sin(k_x) & 0 & 0 \\ e^{-i\phi}\sin(k_y) & 0 & 0 \end{pmatrix}, \tag{8}$$

with $\mathcal{H}_{-1}(\vec{k},\phi) = \mathcal{H}_1^\dagger(\vec{k},\phi)$ and $\rho = 2t_0\mathcal{J}_1(A_0)$. We find that the commutator $\mathfrak{C}(\phi) = \left[\mathcal{H}_1(\vec{k},\phi), \mathcal{H}_{-1}(\vec{k},\phi)\right]$ yields

$$\mathfrak{C}(\phi) = i\rho'(\phi) \begin{pmatrix} 0 & 0 & 0 \\ 0 & 0 & \sin(k_x)\sin(k_y) \\ 0 & -\sin(k_x)\sin(k_y) & 0 \end{pmatrix}, \tag{9}$$

where $\rho'(\phi) = 8[t_0\mathcal{J}_1(A_0)]^2\sin(\phi)$. In the spin space, the commutator is equivalent to a next-nearest neighbour (NNN) DM interaction,

$$\mathfrak{C}(\phi) = \rho'(\phi)\sum_{\langle\langle \ell\ell'\rangle\rangle} \nu_{\ell\ell'}\hat{z}\cdot\vec{S}_\ell \times \vec{S}_{\ell'}, \tag{10}$$

where $\nu_{\ell\ell'} = \pm 1$ for magnon hopping clockwise or counterclockwise on the Lieb lattice. This term obviously breaks time-reversal symmetry. In this way, we have generated a synthetic next-nearest neighbour DM interaction on the Lieb lattice using circularly-polarized laser light as shown in Fig. (1)(a). The effective Hamiltonian is given by

$$\mathcal{H}_{\text{eff}}(\vec{k},\phi) \simeq t_z \mathrm{I}_{3\times 3} + \vec{d}\cdot\vec{\lambda}, \tag{11}$$

where $\mathrm{I}_{3\times3}$ is an identity matrix, $\vec{d} = (d_x, d_y, d_z)$ with $d_x = -2\Delta_0\cos(k_x)$, $d_x = -2\Delta_0\cos(k_y)$, $d_z = \Delta_1(\phi)\sin(k_x)\sin(k_y)$, where $\Delta_0 = t_0\mathcal{J}_0(A_0)$, $\Delta_1(\phi) = 8[t_0\mathcal{J}_1(A_0)]^2\sin(\phi)/\omega$, and $\mathcal{J}_n$ is the Bessel function of order n. In the limit $A_0 \ll 1$ we have $\Delta_0 \approx t_0$ and $\Delta_1(\phi) \approx 2t_0^2A_0^2\sin(\phi)/\omega$. $\vec{\lambda} = (\lambda_x, \lambda_y, \lambda_z)$ are the Gell-Mann matrices which form the pseudospin-1 representation of the SU(2) Lie algebra.

$$\lambda_x = \begin{pmatrix} 0 & 1 & 0 \\ 1 & 0 & 0 \\ 0 & 0 & 0 \end{pmatrix}, \; \lambda_y = \begin{pmatrix} 0 & 0 & 1 \\ 0 & 0 & 0 \\ 1 & 0 & 0 \end{pmatrix}, \; \lambda_z = \begin{pmatrix} 0 & 0 & 0 \\ 0 & 0 & i \\ 0 & -i & 0 \end{pmatrix}. \tag{12}$$

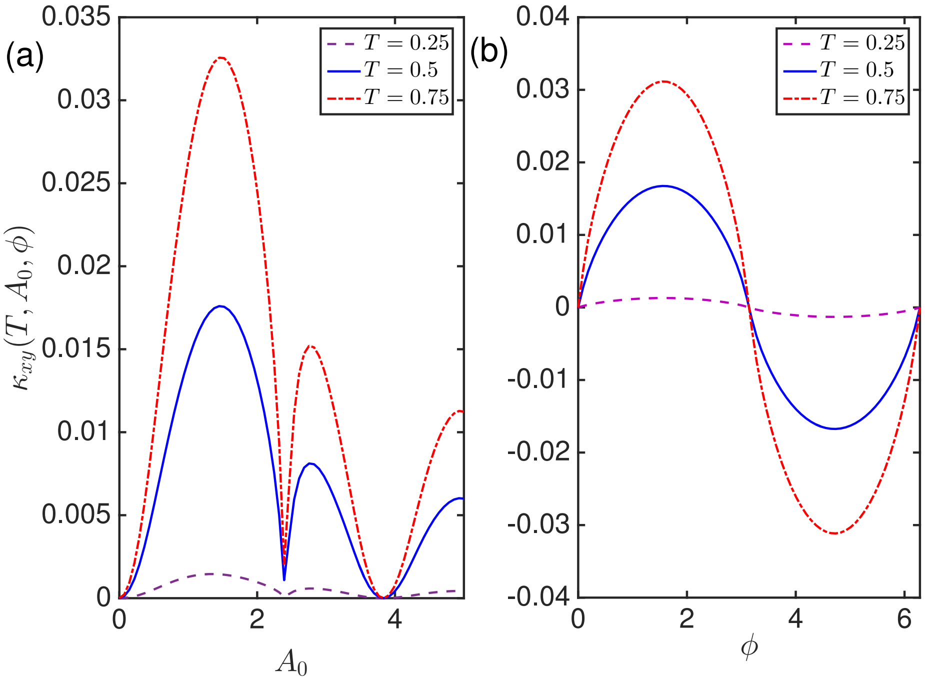

FIG. 3: Floquet tunable thermal Hall conductivity of magnons on the Lieb-lattice ferromagnets. (a) $\kappa_{xy}(T, A_0, \phi)$ vs. A_0 for several values of T at $\phi = \pi/2$. (b) $\kappa_{xy}(T, A_0, \phi)$ vs. ϕ for various T at $A_0 = 1.7$. Here $\hbar\omega = 10J$ in both plots.

The Floquet magnon bands in the zeroth and first order approximation are shown in Fig. (2)(a) and (b) respectively. Expanding the effective Floquet-Bloch magnon Hamiltonian (11) around the **M**-point yields a topologically gapped pseudospin-1 Dirac magnon with the gap $\sim 2\Delta_1(\phi)$. Hence, the system becomes a Floquet magnon Chern insulator. In the vicinity of the **M**-point the effective Floquet-Bloch magnon Hamiltonian yields

$$\mathcal{H}_{\text{eff}}(\mathbf{M} + \vec{q}, \phi) \simeq t_z \mathrm{I}_{3\times 3} + v_\perp \left(q_x\lambda_x + q_y\lambda_y\right) + v_z(\phi)\lambda_z, \tag{13}$$

where $v_\perp \simeq 2t_0$ and $v_z(\phi) \simeq 2t_0^2 A_0^2 \sin(\phi)/\omega$ in the limit $A_0 \ll 1$. We define the Floquet-Bloch Berry curvature for the magnon band α as

$$\begin{aligned}\Omega_\alpha(\vec{k}, \phi) &= -2\mathrm{Im}\big[\, \langle \partial_{k_x}\xi_\alpha(\vec{k}) | \partial_{k_y}\xi_\alpha(\vec{k})\rangle \,\big],\\ &= \left[0, \mp\frac{\vec{d}\cdot\left(\partial_{k_x}\vec{d}\times\partial_{k_y}\vec{d}\right)}{d^3}\right],\end{aligned} \tag{14}$$

with 0 representing the flat band and $d = \sqrt{d_x^2 + d_y^2 + d_z^2}$. The associated Chern number is given by

$$\mathcal{C}_\alpha(\phi) = \frac{1}{2\pi}\int_{\text{BZ}} d^2k\ \Omega_\alpha(\vec{k}, \phi). \tag{15}$$

The Chern number can be calculated analytically [27] and we find $\mathcal{C}_\alpha(\phi) = 0, \mp\mathrm{sgn}(\Delta_1(\phi))$ for the flat and dispersive bands respectively.

Floquet thermal Hall effect on the Lieb lattice. Charge-neutral bosonic quasiparticles such as magnons show a thermal Hall effect when a transverse heat current flows under the influence of a longitudinal temperature gradient, in the presence of a magnetization and an intrinsic DM interaction, as reported experimentally in ferromagnetic insulators [15, 28, 29]. In the current study, circularly-polarized light induces a synthetic tunable DM interaction. However, the distribution of magnons in the system will also be out of equilibrium. Nonetheless, in the high frequency limit, the Bose distribution function is close to equilibrium, hence we can apply linear response theory and obtain the heat current $\mathcal{J}_\alpha^Q = -\sum_\beta \kappa_{\alpha\beta}\nabla_\beta T$, where $\kappa_{\alpha\beta}$ is the thermal conductivity. The transverse

component κ_{xy} is associated with the thermal Hall conductivity given explicitly as [30]

$$\kappa_{xy}(T, A_0, \phi) = -k_B^2 T \int_{\text{BZ}} \frac{d^2k}{(2\pi)^2} \sum_{\alpha=1}^{N} c_2\left(n_\alpha\right) \Omega_\alpha(\vec{k}, \phi), \tag{16}$$

where $n_\alpha = n\big[\epsilon_\alpha(\vec{k})\big] = 1/\big[e^{\epsilon_\alpha(\vec{k})/k_B T} - 1\big]$ is the Bose distribution function close to thermal equilibrium, k_B is the Boltzmann constant, T is the temperature, and $c_2(x) = (1+x)\left(\ln \frac{1+x}{x}\right)^2 - (\ln x)^2 - 2\text{Li}_2(-x)$, with $\text{Li}_2(x)$ being the dilogarithm.

In Fig. (3) we have shown the trends of the Floquet thermal Hall conductivity as a function of the tunable parameters. The plot of $\kappa_{xy}(T, A_0, \phi)$ vs. A_0 is shown in Fig. (3)(a) for several values of T. We see that the Floquet thermal Hall conductivity is oscillating due to the Bessel functions. Therefore, $\kappa_{xy}(T, A_0, \phi)$ can be turned off at the zeros of the Bessel functions and tuned back on away from the zeros. In this way, one can manipulate magnon spin currents in magnetic insulators using circularly-polarized laser light. This is a special feature of breaking time-reversal symmetry by photo-irradiation as opposed to magnetic insulators with intrinsic symmetry breaking perturbations that cannot be tuned. Fig. (3)(b) shows the trend $\kappa_{xy}(T, A_0, \phi)$ vs. ϕ for several values of T. Here, the sign of the Floquet thermal Hall conductivity is changed by varying the phase difference ϕ. The sign change is consistent with the sign change of the lowest band Chern number as ϕ is varied. We note that a sign change is not possible in the NN Lieb lattice ferromagnet with a static NNN DM interaction [9].

---

[1] F. D. M. Haldane, Phys. Rev. Lett. **61**, 2015 (1988).
[2] C. L. Kane and E. J. Mele, Phys. Rev. Lett. **95**, 146802 (2005).
[3] X. -L. Qi and S. -C. Zhang, Rev. Mod. Phys. **83**, 1057 (2011).
[4] M. Z. Hasan and C. L. Kane, Rev. Mod. Phys. **82**, 3045 (2010).
[5] H. Katsura, N. Nagaosa, and P. A. Lee, Phys. Rev. Lett. **104**, 066403 (2010).
[6] L. Zhang et al., Phys. Rev. B **87**, 144101 (2013).
[7] A. Mook, J. Henk, and I. Mertig, Phys. Rev. B **90**, 024412 (2014); Phys. Rev. B **89**, 134409 (2014).
[8] H. Lee, J. H. Han, and P. A. Lee, Phys. Rev. B. **91**, 125413 (2015).
[9] X. Cao, K. Chen, and D. He, J. Phys.: Condens. Matter **27**, 166003 (2015).
[10] S. A. Owerre, J. Phys.: Condens. Matter **28**, 386001 (2016).
[11] S. A. Owerre, J. Appl. Phys. **120**, 043903 (2016).
[12] S. K. Kim et al., Phys. Rev. Lett. **117**, 227201 (2016).
[13] A. Roldán-Molina, A. S. Nunez, and J. Fernández-Rossier, New J. Phys. **18**, 045015 (2016).
[14] R. Chisnell et al., Phys. Rev. Lett. **115**, 147201 (2015).
[15] T. Ideue et al., Phys. Rev. B. **85**, 134411 (2012).
[16] V. J. Emery, Phys. Rev. Lett. **58**, 2794 (1987).
[17] R. T. Scalettar et al, Phys. Rev. B **44**, 770 (1991).
[18] D. Guzmán-Silva et al., New J. Phys. **16**, 063061 (2014).
[19] R. A. Vicencio et al., Phys. Rev. Lett. **114**, 245503 (2015).
[20] S. Taie et al., Sci. Adv. **1**, e1500854 (2015).
[21] S. Mukherjee et al., Phys. Rev. Lett. **114**, 245504 (2015).
[22] F. Diebel et al., Phys. Rev. Lett. **116**, 183902 (2016).
[23] Y. Long and J. Ren, arXiv:1706.01107 (2017).
[24] S. A. Owerre, J. Phys. Commun. **1**, 021002 (2017).
[25] J. Fransson, A. M. Black-Schaffer, and A. V. Balatsky, Phys. Rev. B **94**, 075401 (2016).
[26] K. Nakata, J. Klinovaja, and D. Loss, Phys. Rev. B. **95**, 125429 (2017).
[27] Y. He, J. Moore, and C. M. Varma, Phys. Rev. B **85**, 155106 (2012).
[28] Y. Onose et al., Science **329**, 297 (2010).
[29] M. Hirschberger et al., Phys. Rev. Lett. **115**, 106603 (2015).
[30] R. Matsumoto and S. Murakami, Phys. Rev. Lett. **106**, 197202 (2011); Phys. Rev. B **84**, 184406 (2011).
